# Supplementary material for: The competing effects of racial discrimination and racial identity on the predicted number of days incarcerated in the US: A national profile of Black, Latino/Latina, and American Indian/Alaska Native populations
Source: PLoS One. 2022 Jun 8;17(6):e0268987. doi: 10.1371/journal.pone.0268987 (PMC9176760; doi:10.1371/journal.pone.0268987)
Supplement: S1 Table — (DOCX) [file pone.0268987.s001.docx]

Supplemental Table 1 – Pearson’s correlation coefficient matrix

| **Variables** | **Total**  N=14,728 | **Black**  n=7,445 | **Latino/Latina**  n=6,804 | **American Indian/Alaska Native**  n=479 |
| --- | --- | --- | --- | --- |
| Days incarcerated * Discrimination | r = 0.040  p < 0.0001 | 0.055  <0.0001 | 0.023  0.06 | 0.071  0.12 |
| Days incarcerated * Identity | -0.020  0.01 | -0.22  0.05 | -0.021  0.08 | 0.011  0.82 |
| Days incarcerated * Age rate | 0.839  <0.0001 | 0.850  <0.0001 | 0.827  <0.0001 | 0.809  <0.0001 |
| Days incarcerated * Female | -0.087  <0.0001 | -0.104  <0.0001 | -0.066  <0.0001 | -0.136  0.01 |
| Days incarcerated * Highest grade | -0.018  <0.0001 | -0.039  <0.01 | -0.006  0.64 | -0.015  0.75 |
| Days incarcerated * Alcohol/drug | 0.098  <0.0001 | 0.103  <0.0001 | 0.092  <0.0001 | 0.130  0.01 |
| Discrimination * Identity | -0.039  <0.0001 | -0.067  <0.0001 | -0.026  0.03 | 0.175  <0.01 |
| Discrimination * Age rate | 0.045  <0.0001 | 0.071  <0.0001 | 0.017  0.17 | 0.046  0.31 |
| Discrimination * Female | -0.053  <0.0001 | -0.076  <0.0001 | -0.034  0.01 | 0.011  0.82 |
| Discrimination * Highest grade | 0.058  <0.0001 | -0.069  <0.0001 | -0.295  <0.0001 | 0.061  0.18 |
| Discrimination * Alcohol/drug | 0.169  <0.0001 | -0.054  <0.0001 | 0.170  <0.0001 | 0.144  0.01 |
| Identity * Age rate | -0.028  <0.01 | -0.031  0.01 | -0.026  0.03 | -0.007  0.88 |
| Identity * Female | 0.003  0.69 | -0.003  0.76 | 0.011  0.36 | -0.020  0.66 |
| Identity * Highest grade | -0.018  0.03 | -0.069  <0.0001 | -0.029  <0.0001 | -0.121  0.01 |
| Identity * Alcohol/drug | -0.097  <0.0001 | -0.054  <0.0001 | -0.150  <0.0001 | -0.021  0.64 |
| Age rate * Female | -0.086  <0.0001 | -0.106  <0.0001 | -0.064  <0.0001 | -0.120  0.01 |
| Age rate * Highest grade | -0.019  0.02 | -0.040  <0.01 | -0.007  0.54 | 0.061  0.18 |
| Age rate * Alcohol/drug | 0.105  <0.0001 | 0.112  <0.0001 | 0.095  <0.0001 | 0.134  <0.01 |
| Female * Highest grade | 0.042  <0.0001 | 0.066  <0.0001 | 0.012  0.29 | 0.035  0.45 |
| Female * Alcohol/drug | -0.163  <0.0001 | -0.182  <0.0001 | -0.145  <0.0001 | -0.149  0.01 |
| Highest grade * alcohol/drug | 0.043  <0.0001 | -0.045  <0.01 | 0.119  <0.0001 | -0.76  0.09 |
