# Supplementary material for: The competing effects of racial discrimination and racial identity on the predicted number of days incarcerated in the US: A national profile of Black, Latino/Latina, and American Indian/Alaska Native populations
Source: PLoS One. 2022 Jun 8;17(6):e0268987. doi: 10.1371/journal.pone.0268987 (PMC9176760; doi:10.1371/journal.pone.0268987)
Supplement: S2 Table — (DOCX) [file pone.0268987.s002.docx]

Supplemental Table 2 – Predicted days incarcerated by individual discrimination and identity survey items

|  |  | **Race/ethnicity** | | | | | | | | | | |
| --- | --- | --- | --- | --- | --- | --- | --- | --- | --- | --- | --- | --- |
| **Survey items** |  | **Black**  *n=7,445* | | |  | **Latino/Latina**  *n=6,804* | | |  | **American Indian/**  **Alaska Native**  *n=479* | | |
|  |  | *Predicted days incarcerated*  *(Low score)* | *Predicted days incarcerated*  *(High score)* | *Percentage difference* |  | *Predicted days incarcerated*  *(Low score)* | *Predicted days incarcerated*  *(High score)* | *Percentage difference* |  | *Predicted days incarcerated*  *(Low score)* | *Predicted days incarcerated*  *(High score)* | *Percentage difference* |
| ***Discrimination items***  *(0-4; 0 = low/none, 4 = high discrimination)* |  |  |  |  |  |  |  |  |  |  |  |  |
|  |  |  |  |  |  |  |  |  |  |  |  |  |
| Experienced discrimination in your ability to obtain healthcare |  | 33.6 | 66.2 | 97.0 |  | 14.2 | 20.6 | 45.1 |  | 19.5 | 54.0 | 176.9 |
| Experienced discrimination in how you were treated when you got care |  | 33.0 | 81.6 | 147.2 |  | 14.7 | 28.4 | 93.2 |  | 29.5 | 4.7 | -84.1 |
| Experienced discrimination in public |  | 26.6 | 122.8 | 361.6 |  | 14.4 | 17.1 | 18.7 |  | 20.9 | 58.0 | 177.5 |
| Experienced discrimination in any other situation |  | 24.4 | 177.5 | 627.4 |  | 11.7 | 51.2 | 337.6 |  | 21.1 | 28.4 | 34.6 |
| Been called a racist name |  | 31.1 | 88.1 | 183.3 |  | 14.2 | 26.8 | 88.7 |  | 16.3 | 67.3 | 312.9 |
| Been made fun of, picked on, or threatened because of your race/ethnicity |  | 34.9 | 48.2 | 38.1 |  | 16.6 | 9.1 | -45.2 |  | 21.5 | 5.9 | -72.6 |
|  |  |  |  |  |  |  |  |  |  |  |  |  |
| ***Identity items***  *(0-6; 0 = low/none, 6 = high identity)* |  |  |  |  |  |  |  |  |  |  |  |  |
|  |  |  |  |  |  |  |  |  |  |  |  |  |
| Strong sense of self as a member of your racial/ethnic group |  | 46.9 | 28.4 | -39.4 |  | 22.7 | 8.0 | -64.7 |  | 20.9 | 11.5 | -44.9 |
| Identifies with other members of your racial/ethnic group |  | 50.6 | 24.9 | -50.7 |  | 23.8 | 9.1 | -61.7 |  | 8.0 | 0.1 | -98.8 |
| Most close friends are from your racial/ethnic group |  | 33.9 | 26.5 | -21.8 |  | 13.8 | 11.2 | -18.8 |  | 33.6 | 0.1 | -99.6 |
| Racial/ethnic heritage is important |  | 73.9 | 20.1 | -72.8 |  | 23.1 | 9.8 | -57.6 |  | 32.9 | 0.1 | -99.7 |
| More comfortable in social situations where other members of your racial/ethnic group are present |  | 54.5 | 27.2 | -50.1 |  | 22.7 | 7.3 | -67.8 |  | 17.9 | 4.7 | -73.7 |
| Proud of your racial/ethnic heritage |  | 175.7 | 26.2 | -85.1 |  | 9.6 | 10.8 | 12.5 |  | 1.0 | 0.9 | -10.0 |
| Racial/ethnic background plays a big part of your interaction with others |  | 57.1 | 9.7 | -83.0 |  | 29.7 | 8.5 | -71.4 |  | 9.8 | 7.8 | -20.4 |
| Values and behaviors are shared by people of your racial/ethnic groups |  | 81.7 | 12.3 | -84.9 |  | 41.7 | 5.6 | -86.6 |  | 27.4 | 8.4 | -69.3 |

*Note: Models adjusted for age rate, sex, highest grade completed, and alcohol/drug use*
