# Supplementary material for: The competing effects of racial discrimination and racial identity on the predicted number of days incarcerated in the US: A national profile of Black, Latino/Latina, and American Indian/Alaska Native populations
Source: PLoS One. 2022 Jun 8;17(6):e0268987. doi: 10.1371/journal.pone.0268987 (PMC9176760; doi:10.1371/journal.pone.0268987)
Supplement: Attachment — Submitted filename: Review of The competing effects of racial discrimination and racial identity on the predicted number of days incarcerated in the US.docx [file pone.0268987.s003.docx]

Review of The competing effects of racial discrimination and racial identity on the predicted

number of days incarcerated in the US

General comments

As I read the definition of “discrimination,” the authors’ description of it seems imprecise. As they operationalize it, it is not a measure of discrimination per se, but a measure of “number of types of discrimination ever encountered and recorded in the self-report.” I am not sure that I agree with the decision to disregard the data in the survey on the frequency with which discrimination was encountered. In revision, I think the authors should have to both: a. justify this decision and b. report on analyses in which they created a variable(s) that took account of the frequency data. This comment is based on my belief that people who experience discrimination “all the time” are likely to react in different ways than those who experience it “almost never.” The authors may also want to explore whether one particular type of discrimination is a better predictor. After all, with an N of 14 thousand, such analyses should be possible.

Similar explorations should be made and reported with different ways to define racial identity. Number of types of identification ignores the issue of strength of identity. And it is also of interest to know if one particular kind of identification is more or less associated with incarceration.

Analytically, if the dependent variable is number of days of incarceration, then instead of treating age as a confounder, it should be used to create a rate dependent variable that is defined as “number of days incarcerated divided by numbers of years at risk of being incarcerated.” This is because I expect that self-reports of discrimination; racial identity; and number of days incarcerated are all correlated with age. This is certainly true for days of incarceration. The other variables are likely related because of one or both of the following: a. generations have different experiences and beliefs (see Mannheim’s analysis of generations) and/or b. life stage factors. This seems to me to be a very serious error.

I am skeptical of the unvalidated claim that 86% of the sample reported zero days of incarceration. First, these are self-reported data, and people may be reluctant to disclose criminal histories. Second, even if they are willing to disclose, they might minimize it; or they might say “never incarcerated” in order to avoid follow up questions (depending on skip patterns) and shorten the interview. To be credible, this figure should be supported by comparisons with other studies (such as other surveys) that do not focus on alcoholism but perhaps on criminal victimization and other surveys like some NORC has done.

In an analysis such as this, reporting on the correlations among the main analytic data (probably for total, and within racial categories) is needed. Particularly concerning is the possibility that racial identity and racial discrimination experiences could conceivably be very high.

I think that the Conclusions should include a strong statement about the much higher rates of “ever incarcerated” among AIAN. Given the many limitations that accrue to simply reporting that you were ever arrested in job applications and other critical processes, this is of enormous impact.

Discussion and Conclusions discuss structural racism a lot. This makes the analytic failure to include any measures of structural racism stand out as a major defect and limitation. (See below under “important additional comments.”

Important additional comments

1. Perhaps the title should indicate the restricted nature of the sample that was analyzed by adding “among Black, Latino/Latina, and AIAN individuals.” Although the exclusion of whites is not problematic given the theoretical framing and the realities of oppression, the exclusion of Asian Americans is an important limitation and should be noted in the title (I think) and should be justified in Methods.
2. Limitations should include the inability to analyze how locality or workplace influenced incarceration. Measures of structural racism like racial/ethnic population densities, racial/ethnic residential and occupational segregation are almost certainly associated with probability of getting arrested, and likely with both racial discrimination and racial identity.

Minor Comments

1. line 2 “bared” is misleading. Probably “borne” would be best
2. line 5 – 6 “remain…remains” I will stop text editing at this point. It is clear that serious text editing is needed.
3. l 9 I am not sure “disenfranchisement” is the best term for this.
4. p. 5 l 6 – 7: Here or in Methods, the variable that was used to limit the sample to Black, Latino/Latina, and AIAN individuals should be described.
5. Table 1 and first paragraph of Results: Important to state whether mean days incarcerated is for the total analytic sample or only for the subset who reported non-zero on this variable.
6. p. 13 -14: I am not sure that the claim about structural racism in the following sentence is justified by the data (although I think it fully justified in reality): “This study supports the notion that incarceration is driven, in part, by the mechanisms of structural racism and personal discriminatory acts towards racial/ethnic minorities.” I say this because I see no measures of structural racism in the set of independent variables.
