# Supplementary material for: The competing effects of racial discrimination and racial identity on the predicted number of days incarcerated in the US: A national profile of Black, Latino/Latina, and American Indian/Alaska Native populations
Source: PLoS One. 2022 Jun 8;17(6):e0268987. doi: 10.1371/journal.pone.0268987 (PMC9176760; doi:10.1371/journal.pone.0268987)
Supplement: Attachment — Submitted filename: PLOS discrimination_Response to reviewers.docx [file pone.0268987.s004.docx]

We thank the journal editors and our two reviewers for the opportunity to revise our manuscript. We received invaluable insight from our reviewers about study design, more accurately defining discrimination and identity, and further exploring the influence of discrimination on incarceration risk. We believe our manuscript has been substantially improved as a result of the thoughtful consideration put into each review and is now suitable for publication in *PLOS One*. We have responded to each comment in detail below, and have submitted a revised manuscript with changes tracked in MS Word.

**Reviewer 1**

**General comments**

**1. The authors conducted a study of racial discrimination, racial identity, and incarceration risk in a national US sample of Black, Latino/Latina, and American Indian/Alaska Native individuals. Overall, the study is well written, interesting, and will be an important contribution to the literature. The methods are appropriate, thorough, and well-described. I only recommend some minor edits to improve clarity and language.**

Response: Thank you for your critical feedback and positive support for our research. We have responded to each of your comments below.

**2. Overall, I recommend not using the term "Blacks", and instead using "Black populations", "Black individuals", or "Black participants".**

Response: We appreciate this feedback as we are constantly striving to be conscious of the language we use to describe disparities. We have gone through the manuscript and edited how we describe groups, in line with using “Black individuals” or “American Indian/Alaska Native respondents”.

**Methods**

**3. When you discuss the survey procedures and design effects, I'd recommend a sentence on what the clusters and strata represent (like groups of counties, cities, etc.).**

Response: This is a good opportunity to provide more information about the survey design to our readers. The probability sampling procedure of NESARC-III is complex, including primary sampling units, secondary sampling units, segments, dwelling units, and respondents. We added two sentences in our methods section outlining the purpose and process of oversampling in high-minority areas, and direct the readers to the NESARC technical documentation for further information.

**Results**

**4. For the last sentence that "These trends were consistent between racial/ethnic groups, and were strongest among AIAN", I would revise to drop the "and were strongest among AIAN", as it looks like Black and Latinx participants had the strongest association between discrimination and incarceration.**

Response: Please see our response to Comments #7 and #8. Our definitions for discrimination and identity have changed to include the full information available on the frequency of discrimination exposure and the magnitude of agreement with identity statements. As such, our regression estimates have changed moderately and our results section has been updated, including the sentence described in the comment above.

**Discussion**

**5. Some discussion on how racial identity buffers against internalized racism is recommended.**

Response: Better understanding the mechanisms underlying the buffering characteristics of racial identity is an important future research question that came out of this current study. For this study, we believe that speculating on *how* identity may be operating to suppress the effect of discrimination exposure is beyond the scope of what we measured. However, we did add several sentences to our discussion section outlining how our findings align with previous research addressing the intersection of identity and discrimination.

**Limitations**

**6. The distinction between jails and prisons noted in the limitations is important; I recommend an additional sentence on how racial discrimination may differ between them.**

Response: We have added a sentence about the importance of considering how racial discrimination may vary between the two locales.

**Reviewer 2**

**General comments**

**7. As I read the definition of “discrimination,” the authors’ description of it seems imprecise. As they operationalize it, it is not a measure of discrimination per se, but a measure of “number of types of discrimination ever encountered and recorded in the self-report.” I am not sure that I agree with the decision to disregard the data in the survey on the frequency with which discrimination was encountered. In revision, I think the authors should have to both: a. justify this decision and b. report on analyses in which they created a variable(s) that took account of the frequency data. This comment is based on my belief that people who experience discrimination “all the time” are likely to react in different ways than those who experience it “almost never.”**

Response: Thank you for this very helpful comment. We agree that our original definition of discrimination was imprecise – our single category of ‘any’ discrimination included a wide range of experiences, from almost never to all the time. This variable failed to capture what we are trying to measure, which is the frequency, or magnitude, of exposure to discriminatory behavior.

We replaced the original ‘any/none’ variable with a new variable that takes into account the frequency of discrimination exposure. The new composite scale is based on six survey questions, each with response options of 0 (never) to 4 (very often), resulting in a single scale of 0 to 24. Scores at the higher end of the spectrum result from multiple responses endorsing often or very often exposure. This new approach allows for more granularity in measuring discrimination, as higher scores reflect a greater frequency of discrimination.

**8. Similar explorations should be made and reported with different ways to define racial identity. Number of types of identification ignores the issue of strength of identity.**

Response: Our original racial/ethnic identity scale was similar to the original discrimination scale, in that we defined ‘any’ positive identity as any respondent that somewhat agreed, agreed, or strongly agreed with each statement. The original variable was based on eight survey questions and the scale ranged from 0 to 8. However, applying the same logic as the reviewer raised above in Comment #7, experiences of individuals who ‘somewhat agree’ are likely different than those who ‘strongly agree’. Therefore we reconstructed our identity variable to reflect the magnitude, or strength, of affirming responses. The revised identity scale now ranges from 0 to 48, with higher scores reflecting a greater magnitude of agreement with each question.

**9. The authors may also want to explore whether one particular type of discrimination is a better predictor. After all, with an N of 14 thousand, such analyses should be possible.
And it is also of interest to know if one particular kind of identification is more or less associated with incarceration.**

Response: This is a great point, as our composite discrimination and identity scales do not capture the influence of each individual sub-item on incarceration. We created a Supplemental Table 1, which outlines the relationship between each survey item and incarceration. Specifically, we derived the predicted days incarcerated for the lowest and highest scores for each question, and also reported the percentage difference in days. For example, among Black respondents, the predicted days incarcerated for those with no experience with discrimination in healthcare settings was 33.6, whereas the predicted days incarcerated for those that have experienced discrimination in healthcare ‘very often’ was 66.2, or a 97.0% increase in days incarcerated between ‘no’ discrimination and ‘very often’ discrimination.

**Title**

**10. Perhaps the title should indicate the restricted nature of the sample that was analyzed by adding “among Black, Latino/Latina, and AIAN individuals.” Although the exclusion of whites is not problematic given the theoretical framing and the realities of oppression, the exclusion of Asian Americans is an important limitation and should be noted in the title (I think) and should be justified in Methods.**

Response: We have changed our title to state specifically the groups included in this study, and have added text in the methods section about how we arrived at our final analytic sample. Our new title is “The competing effects of racial discrimination and racial identity on the predicted number of days incarcerated in the US: A national profile of Black, Latino/Latina, and American Indian/Alaska Native populations”.

**Introduction**

**11. Line 2 “bared” is misleading. Probably “borne” would be best**

Response: We have changed ‘bared’ to ‘borne’.

**12. Line 5 – 6 “remain…remains” I will stop text editing at this point. It is clear that serious text editing is needed.**

Response: We appreciate the opportunity to make our writing as clear as possible for our readers. In this particular case, we have respectfully kept the word ‘remain’, as it refers to the plural noun ‘populations’. We have reviewed our manuscript thoroughly for grammatical errors and typos, and have edited sentence structure to be as clear and correct as possible.

**13. Line 9 I am not sure “disenfranchisement” is the best term for this.**

Response: We have changed ‘one form of disenfranchisement’ to ‘one factor in particular’.

**Methods**

**14. Analytically, if the dependent variable is number of days of incarceration, then instead of treating age as a confounder, it should be used to create a rate dependent variable that is defined as “number of days incarcerated divided by numbers of years at risk of being incarcerated.” This is because I expect that self-reports of discrimination; racial identity; and number of days incarcerated are all correlated with age. This is certainly true for days of incarceration. The other variables are likely related because of one or both of the following: a. generations have different experiences and beliefs (see Mannheim’s analysis of generations) and/or b. life stage factors. This seems to me to be a very serious error.**

Response: This is a great point and a methodological oversight on our part. While we still report on age groups in our descriptive Table 1, we have replaced age group with a rate dependent variable in our predictive models.

**15. I am skeptical of the unvalidated claim that 86% of the sample reported zero days of incarceration. First, these are self-reported data, and people may be reluctant to disclose criminal histories. Second, even if they are willing to disclose, they might minimize it; or they might say “never incarcerated” in order to avoid follow up questions (depending on skip patterns) and shorten the interview. To be credible, this figure should be supported by comparisons with other studies (such as other surveys) that do not focus on alcoholism but perhaps on criminal victimization and other surveys like some NORC has done.**

Response: Recognizing possible bias in responses to sensitive questions about incarceration history is critical for this study, and not including this as a limitation was an oversight. We have included a paragraph in our limitations section outlining how the possibility of desirability bias could affect the reported estimates of incarceration. In addition, we compared our findings to BJS jail admission data, and concluded that while NESARC-III is imperfect, it is a useful and validated tool to estimate population characteristics, including experiences of incarceration.

**16. In an analysis such as this, reporting on the correlations among the main analytic data (probably for total, and within racial categories) is needed. Particularly concerning is the possibility that racial identity and racial discrimination experiences could conceivably be very high.**

Response: This was helpful for us to think through the complex relationships between all of our analytic variables. We created a new Supplemental Table 2 which includes all of the information from a Pearson’s correlation matrix, for the total sample and stratified by race/ethnicity.

**17. The variable that was used to limit the sample to Black, Latino/Latina, and AIAN individuals should be described.**

Response: We have expanded our description of the race/ethnicity variable we used. NESARC=III provides a single variable that includes pre-constructed categories of race and ethnicity. Please also see our response to Comment #10, where we expanded our description of each racial/ethnic category available in NESARC-III.

**Results**

**18. Table 1 and first paragraph of Results: Important to state whether mean days incarcerated is for the total analytic sample or only for the subset who reported non-zero on this variable.**

Response: We appreciate the opportunity to clarify how we are reporting the study results. The mean days incarcerated is for the total sample, and we have clarified this accordingly in the Results section.

**Discussion**

**19. I think that the Conclusions should include a strong statement about the much higher rates of “ever incarcerated” among AIAN. Given the many limitations that accrue to simply reporting that you were ever arrested in job applications and other critical processes, this is of enormous impact.**

Response: Thank you for this comment – we had not discussed this impactful finding in our original submission but we agree that it deserves attention in our revised discussion section. We have added several sentences and three new citations summarizing the impact of such high rates of incarceration among American Indian/Alaska Native respondents. We also brought to our reader’s attention the juxtaposition of Black respondents having the highest mean number of days incarcerated (78 days), while AI/AN respondents had the highest proportion of any lifetime incarceration (26%).

**20. Discussion and Conclusions discuss structural racism a lot. This makes the analytic failure to include any measures of structural racism stand out as a major defect and limitation.**

Response: We value the opportunity to clarify for our readers how a discussion of structural racism fits in to the overall narrative of race, discrimination, and incarceration. We could not identify any variables within the NESARC dataset that closely resembled anything like structural racism. NESARC does not include a variable for state, which would allow researchers to identify other external state-level data that would serve as a proxy indicator of system-level discrimination and racism, like residential segregation, health provider shortage areas, or state-wide financial estimates of disproportionate loan/mortgage rejections among poor and racial minority groups, for example. At the same time, we believe that incarceration itself is a form of structural racism. We therefore feel that it is our responsibility to engage in a conversation about structural racism, and that this conversation is within the scope of our findings about racial discrimination and incarceration. While not directly measured or tested in this study, structural racism is the language that we choose to use when contextualizing the mass incarceration epidemic.

**21. I am not sure that the claim about structural racism in the following sentence is justified by the data (although I think it fully justified in reality): “This study supports the notion that incarceration is driven, in part, by the mechanisms of structural racism and personal discriminatory acts towards racial/ethnic minorities.” I say this because I see no measures of structural racism in the set of independent variables.**

Response: We agree that this sentence in particular is worded in a way that one may assume we directly tested indicators of structural racism. Even in light of our defense of our discussion around structural racism (see Comment #20), we have removed this sentence to make our study design and focal independent variables as clear as possible.

**Limitations**

**22. Limitations should include the inability to analyze how locality or workplace influenced incarceration. Measures of structural racism like racial/ethnic population densities, racial/ethnic residential and occupational segregation are almost certainly associated with probability of getting arrested, and likely with both racial discrimination and racial identity.**

Response: We have included a new paragraph in our Limitations section that brings to attention the lack of geographic data in NESARC-III, including state. At the very least, access to data about which state the survey respondent resides in would allow for the use of external, state-level indicators of social, environmental, occupational, and economic determinants of health.
