# Supplementary material for: The competing effects of racial discrimination and racial identity on the predicted number of days incarcerated in the US: A national profile of Black, Latino/Latina, and American Indian/Alaska Native populations
Source: PLoS One. 2022 Jun 8;17(6):e0268987. doi: 10.1371/journal.pone.0268987 (PMC9176760; doi:10.1371/journal.pone.0268987)
Supplement: Attachment — Submitted filename: PLOS response to reviewers, round 3.docx [file pone.0268987.s005.docx]

**Comments from the editor**

ABSTRACT

**Comment 1: Page 2, Lines 11-12: change ‘discrimination exposure’ to ‘racial discrimination exposure’.**

Response: We have made this change.

**Comment 2: Page 2, Line 15: What AI/IN stand for? please spell out these acronyms and other acronyms on their first appearance.**

Response: Thank you for catching this. We have spelled out the acronym for American Indian/Alaska Native at first use, which is in the second sentence of the abstract.

**Comment 3: Page 2, Line 19: The conclusion should be based on / refer to the results, which show that Racial discrimination and racial identity are associated with incarceration.**

Response: Thank you for helping us better organize our abstract conclusion. We have reframed this paragraph to restate our findings about discrimination and identity. At the same time, we respectfully feel that it is within the scope of this study to reference mass incarceration and racism as drivers of disparities. Later in the paper, our discussion uses these concepts to contextualize increased risk for involvement in the criminal justice system among underrepresented groups. As an epidemiologic study through a public health lens, we believe that failing to articulate the upstream factors affecting incarceration would be a disservice to our audience. In addition, please see Comment 12 below (Reviewer 2). The sentiment of this feedback was that the abstract conclusion should focus more on these same issues we raised in the discussion section, including structural determinants of health and their effect on incarceration and community health.

INTRODUCTION

**Comment 4: Page 3: Please revise ‘upwards of 10 times higher’. It should be either ‘upwards’ or ‘higher’.**

Response: We have rephrased this sentence to read, “nearly 10 times higher”

**Comment 5: Page3, lines 21-22: Please provide a citation to support the statement: Eighty-five percent of Blacks and 51% of Latino/Latinas study participants in California reported ever being treated unfairly because of their race/ethnicity, compared of Whites participants.’**

Response: We have removed this statement.

METHODS

**Comment 6: Page7, lines-9-10: Please check the statement: ‘educational attainment (less than high school, high school completion, some college, or college graduate), what’s the difference between ‘some college’ and or ‘college graduate’? These could be merged in one category.**

Response: Thank you for the opportunity to clarify our methods and variable creation. We agree that there are multiple ways to combine and rearrange this education variable. In this case, there is an important distinction between ‘some college’ and ‘college graduate’, such that the former refers to some experience with college coursework but did not finish, while the latter refers to those who graduated with a bachelor’s degree. College completion in particular has multiple implications for employment prospects and income, and may be a proxy indicator for overall economic stability. For these reasons, we have respectfully maintained the separation between ‘some college’ and ‘college graduate’.

**Comment 7: Page 7, lines11-12:  Please refer to your statement: “We also included a rate dependent age variable, defined as the number of days incarcerated divided by the number of years at risk of being incarcerated.” What is 'rate dependent age variable? It could be rate of something depending on age. Based on the definition given in the next sentence, it could be named as 'incarceration age rate' or 'incarceration risk rate'.**

Response: This is a great question and we appreciate the chance to clarify the definition of all of our study variables. We added this variable to our analysis in response to our second round of peer review. One reviewer specifically suggested that we include this variable, named ‘rate dependent age variable’, and we defined it exactly as recommended by the reviewer. Given that both reviewers have provided two separate rounds of feedback, and both are currently satisfied with the methods and variables as they are (below), we are reluctant to make any further changes that may contradict our response to their previous review.

LIMITATIONS

**Comment 8: Page 15, line13: BJS 2019 report. Could you please spell out what BJS stands for? Also provide a citation for the report.**

Response: We have included the acronym ‘BJS’ at the first use of the phrase ‘Bureau of Justice Statistics’. We have also made it clear that the citation for the BJS report (included in two sentences) is for citation #33, or Zeng et al.

REFERENCES:

**Comment 9: Please report journal names in the abbreviated form, where available.**

Response: We have changed the journal names to the abbreviated form.

**Reviewer #1**

**Comment 10: The authors have successfully addressed my concerns. I have no further recommendations regarding the manuscript.**

Response: Thank you for your thoughtful and careful consideration of our manuscript.

**Reviewer #2**

**Comment 11: This is an excellent revision and an important paper.**

Response: Thank you for your time and energy put into this critical peer review.

**Comment 12: I have one suggestion about how to improve the Abstract, though I view it as optional for the authors. As currently written, the Conclusions section of the abstract seems so general as to convey little meaning. I think the last paragraph of conclusions in the text has excellent suggestions for interventions, and these should be the focus of the conclusions in abstract. This will also, in my opinion, lead to more people reading the paper.**

Response: Thank you for your positive feedback about our discussion of structural drivers of inequity. Please see our response to Comment 3 by the journal editor. We have kept most of the language used to contextualize our findings and reflect the points we raise in the discussion section about systemic racism and discrimination. At the same time, we are striving to balance this contextual narrative with restating the study findings and keeping the abstract as succinct as possible, as per the journal editor’s comment referenced above.
