# Supplementary material for: The competing effects of racial discrimination and racial identity on the predicted number of days incarcerated in the US: A national profile of Black, Latino/Latina, and American Indian/Alaska Native populations
Source: PLoS One. 2022 Jun 8;17(6):e0268987. doi: 10.1371/journal.pone.0268987 (PMC9176760; doi:10.1371/journal.pone.0268987)
Supplement: Attachment — Submitted filename: PLOS response to reviewers, round 4.docx [file pone.0268987.s006.docx]

Comments from the editor

**Comment #1: In your last reply to the AE/Reviewers, you have stated that 'some college' category of the educational attainment refers to those who had "some experience with college coursework but did not finish". If so then this category could be better reported as 'college student', if studying at the time of completing the survey or 'college dropouts' if they had left the college without completing/graduating. Please address this issue in the text as well as Tables including supplemental material.**

Response: We appreciate the opportunity to clarify how this variable was created and defined. Please see our response to Comment #4. We have added several sentences to the methods section more clearly outline the specific survey response options that were included in our condensed analytic variable. We have also changed the names of the variable levels to more accurately reflect the highest level of education attained by each survey respondent. We updated Table 1 with the new labels. Table 2 and the supplemental material did not reference the education variable levels.

**Comment #2: Tables 1 and 2: Please shift the column 'Total' as the last column on the right hand side because the focus of the study is on the ethnic/racial groups and not on the total/aggregate of the groups.**

Response: We have shifted the total column to the right hand side for both Table 1 and Table 2.

**Comment #3: Three categories of tertiles: low, mid, and high have been used/reported in the text, table 2 and figures 1A-D. Could you please report in the methods section, how low, mid and high tertiles were determined and what values are covered by each of these three tertile categories?**

Response: We have added a paragraph in the methods section defining how tertiles are created based on the distributions of the identity and discrimination scores around the 33^rd^ and 66^th^ percentiles. We have also included the range of identity and discrimination scores for each of the tertile groups.

Reviewer 2

**Comment #4: I have one minor and optional suggestion for a wording change: When you define the variable about education, the precise definition of "some college" and "college graduate" remains unclear. The issues are that one can graduate from a junior college after 2 years; and that college graduate also have some college. So it might be useful to say what you said in the response note, that college grads means undergraduate degree.**

Response: We appreciate the opportunity to clarify how we are defining education, as we agree that the current wording is confusing. While education is not a focus of this study, it acts as an important confounder in our models and we believe it is important for readers to understand how this variable was constructed. We have added text in the methods section defining each of the survey options that we combined to create the new condensed analytic variable. We have also edited the labels for each level to more clearly articulate the highest level of education attained. The new labels are 1) less than high school, 2) high school completion or GED, 3) some college but did not graduate, or received an associate’s or technical degree, and 4) college completion, bachelor’s degree or higher.

Reviewer 3

**Comment #5: Page 7, line 18, “We grouped discrimination scores (0-6) and identity scores (0-8)” should be revised as “We grouped discrimination scores (0-24) and identity scores (0-48)”.**

Response: We have corrected this typo.

**Comment #6: Page 13, Supplemental Tables 1 and 2 are mentioned, but nowhere can find them.**

Response: Supplemental tables 1 and 2 were included as attachments in the PLOS online application portal. We followed specific instructions not to embed supplemental material in the main document text. I can confirm that they are also included as attachments in this current resubmission. If the supplemental tables do not appear on your end again, please reach out to the academic editor.

**Comment #7: Are there any correlations and/or interactions between racial discrimination and racial identity?**

Response: This is a great question and we appreciate your interest in our research. We believe that testing for correlation or an interaction between identity and discrimination is beyond the scope of the current study, but is very much of interest to us for future research endeavors. For this study, we are specifically interested in the main effects of identity and discrimination on incarceration in a fully adjusted model. Testing the hypothesis that the effect of identity on incarceration is conditional on the value of discrimination would be a valuable contribution to the social sciences and criminal justice literature. At the same time and respectfully, we have opted to make no changes to our statistical model and consider your question about alternative methods as positive motivation to continue this line of research.
